# Draft Genome Sequences of Vancomycin-Susceptible *Staphylococcus aureus* Related to Heterogeneous Vancomycin-Intermediate *S. aureus*

**DOI:** 10.1128/genomeA.01033-14

**Published:** 2014-10-09

**Authors:** Thiruvarangan Ramaraj, Stephanie A. Matyi, Anitha Sundararajan, Ingrid E. Lindquist, Nicolas P. Devitt, Faye D. Schilkey, Reena Lamichhane-Khadka, Peter R. Hoyt, Joann Mudge, John E. Gustafson

**Affiliations:** a National Center for Genome Resources (NCGR), Santa Fe, New Mexico, USA; b Department of Biochemistry and Molecular Biology, Oklahoma State University, Stillwater, Oklahoma, USA; c Department of Biology, New Mexico State University, Las Cruces, New Mexico, USA; d Department of Biology, Saint Mary’s College, Notre Dame, Indiana, USA

## Abstract

We report the draft genome sequences of three vancomycin-susceptible methicillin-resistant *Staphylococcus aureus* strains. *S. aureus* strain MV8 is a sequence type 8 (ST-8) staphylococcal cassette chromosome *mec* element type IV (SCC*mec* IV) derivative, while the other two strains (*S. aureus* MM25 and MM61) are ST-5 SCC*mec* II strains. MM61 is also closely related to the heterogeneous vancomycin-intermediate *S. aureus* strain MM66.

## GENOME ANNOUNCEMENT

Here, we report the draft genome sequences of the methicillin-resistant *Staphylococcus aureus* (MRSA) strains MM25, MM61, and MV8 (Table 1). These strains, along with the heterogeneous vancomycin-intermediate *S. aureus* (hVISA) strain MM66 (1), were isolated from hospitals in Las Cruces, New Mexico (2). The SmaI chromosomal DNA pulsed-field gel electrophoresis patterns of MM61 and MM66 demonstrated that these strains are clonal (2).

**TABLE 1:** Draft genome information

Strain	Coverage (×)^*a*^	No. of scaffolds (≥1 kb)	Draft genome size (Mb)	No. of RAST predicted features	Accession no.
MM25	621	57	2.89	2,734	CCCE000000000
MM61	614	78	2.96	2,815	CCCA000000000
MV8	686	81	3.03	2,816	CCCL000000000

a Based on a genome size estimate of 3 Mbp.

Genomic libraries were constructed using the Phusion Illumina genomic DNA library protocol (average insert size, 380 bp) and sequenced with the Illumina Genome Analyzer II 90-bp paired-end reads. The raw sequence reads were then screened for sequencing artifacts and ΦX contamination. *De novo* assemblies were generated using filtered sequence reads utilizing the ABySS version 1.3.7 assembler (3). The GapCloser version 1.12-r6 module from the Short Oligonucleotide Analysis Package (SOAP) (http://soap.genomics.org.cn/) (4) was used to attempt to resolve the N-spacers introduced in the assembly scaffolding process. After the gap filling process, the percentage of gaps was <0.03% per strain, and scaffolds ≥1 kb were retained in the final assembly. An assembly assessment for validation was performed by mapping high-quality Illumina reads back to the assembly using Burrows-Wheeler Aligner (BWA) (5). A high percentage of reads mapped uniquely to the assemblies in each strain (>95% uniquely mapped back, with >90% properly paired for all strains), validating the *de novo* assembly process. Gene prediction and annotation of final assemblies were carried out using RAST (6), incorporating the Glimmer algorithm (7, 8).

The G+C content of all strains (~33%) was similar to that of sequenced *S. aureus* strains (9), as determined by GAEMR evaluation (http://www.broadinstitute.org/software/gaemr/). Sequence analysis revealed that MM25 and MM61 are from multilocus sequence type 5 (ST-5) (http://saureus.mlst.net/) and possess a staphylococcal chromosome cassette *mec* element type II (SCC*mec* II) (http://www.ccrbtyping.net/), while strain MV8 is from ST-8 and harbors SCC*mec* IV. Out of the 152 MRSA strains analyzed in Delgado et al. (2), 123 were resistant to ciprofloxacin, clindamycin, and erythromycin. The draft genomes of all three strains revealed the presence of *ermA*, which supports clindamycin and erythromycin resistance (10). Fluoroquinolone resistance in *S. aureus* is mediated by mutations in the genes encoding DNA gyrase (*gyrA*) or topoisomerase IV (*grlA*) subunits (11–13). MM25 and MM61 harbor *gyrA* and *grlA* mutations that lead to amino acid alterations in GyrA (S84L) and GrlA (S80F), respectively. MM25 also has another mutation in *grlA* leading to an alteration in GrlA (E84G). MM61 also harbors *tetM*, which encodes tetracycline resistance (14).

Vancomycin-intermediate *S. aureus* (VISA) strains, including hVISA, emerge *in vivo* or *in vitro* following vancomycin exposure, and these mutants often possess chromosomal mutation(s) that lead to altered cell wall metabolism (15). None of the three MRSA draft genomes contained the VISA-associated *graS* mutation previously found in MM66 and MM66 derivatives exhibiting altered vancomycin susceptibility (1). hVISA, VISA, and vancomycin-susceptible related strain sets (e.g., MM66, MM66 derivatives, and MM61) are routinely exploited to decipher mutations associated with *S. aureus* vancomycin-intermediate resistance mechanisms (15).

### Nucleotide sequence accession numbers.

These draft genome projects have been deposited at DDBL/EMBL/GenBank under the accession numbers described in Table 1.
